# High frequency of silent brain infarcts associated with cognitive deficits in an economically disadvantaged population

**DOI:** 10.6061/clinics/2017(08)04

**Published:** 2017-08

**Authors:** Paula Squarzoni, Jaqueline H. Tamashiro-Duran, Fabio L.S. Duran, Claudia C. Leite, Mauricio Wajngarten, Marcia Scazufca, Paulo R. Menezes, Paulo A. Lotufo, Tania C.T.F. Alves, Geraldo F. Busatto

**Affiliations:** I Departamento de Psiquiatria, Instituto de Psiquiatria (IPQ), Hospital das Clinicas HCFMUSP, Faculdade de Medicina, Universidade de Sao Paulo, Sao Paulo, SP, BR; II Departamento de Radiologia e Oncologia, Faculdade Medicina FMUSP, Universidade de Sao Paulo, Sao Paulo, SP, BR; III Instituto do Coracao (InCor), Hospital das Clinicas HCFMUSP, Faculdade de Medicina, Universidade de Sao Paulo, Sao Paulo, SP, BR; IV Departamento de Medicina Preventiva, Faculdade de Medicina FMUSP, Universidade of Sao Paulo, Sao Paulo, SP, BR; V Centro de Pesquisa Clinica e Epidemiologica, Faculdade de Medicina FMUSP, Universidade de Sao Paulo, Sao Paulo, SP, BR

**Keywords:** Framingham Coronary Heart Disease Risk, Ageing, Educational Level, Cognition, Silent Brain Infarction

## Abstract

**OBJECTIVE:**

Using magnetic resonance imaging, we aimed to assess the presence of silent brain vascular lesions in a sample of apparently healthy elderly individuals who were recruited from an economically disadvantaged urban region (São Paulo, Brazil). We also wished to investigate whether the findings were associated with worse cognitive performance.

**METHODS:**

A sample of 250 elderly subjects (66-75 years) without dementia or neuropsychiatric disorders were recruited from predefined census sectors of an economically disadvantaged area of Sao Paulo and received structural magnetic resonance imaging scans and cognitive testing. A high proportion of individuals had very low levels of education (4 years or less, n=185; 21 with no formal education).

**RESULTS:**

The prevalence of at least one silent vascular-related cortical or subcortical lesion was 22.8% (95% confidence interval, 17.7–28.5), and the basal ganglia was the most frequently affected site (63.14% of cases). The subgroup with brain infarcts presented significantly lower levels of education than the subgroup with no brain lesions as well as significantly worse current performance in cognitive test domains, including memory and attention (*p*<0.002).

**CONCLUSIONS:**

Silent brain infarcts were present at a substantially high frequency in our elderly sample from an economically disadvantaged urban region and were significantly more prevalent in subjects with lower levels of education. Covert cerebrovascular disease significantly contributes to cognitive deficits, and in the absence of magnetic resonance imaging data, this cognitive impairment may be considered simply related to ageing. Emphatic attention should be paid to potentially deleterious effects of vascular brain lesions in poorly educated elderly individuals from economically disadvantaged environments.

## INTRODUCTION

Brain infarcts and lacunae are common in elderly populations and may be easily detected by high-resolution magnetic resonance imaging (MRI). These lesions are typically associated with poor cognitive function and a loss of the ability to perform daily living activities; they also predict a future risk of dementia 1. According to many recent large-scale MRI studies of elderly populations, a considerable proportion of these brain infarcts remains undetected due to the absence of overt clinical impairments 2-4. In contrast to the traditional view that cardiovascular diseases result from a combination of genetic, lifestyle and physiological risk factors, a recent trend has incorporated the social determinants of health as an additional, critical dimension of cardiovascular risk 5. Education is the most frequently used indicator of socioeconomic position in the United States and was the predictor with the highest significance in determining cardiovascular disease outcomes 5.

Elderly subjects with very low levels of education and disadvantageous socioeconomic conditions, which are common in many low- and middle-income countries, present increased rates of cardiovascular risk factors, stroke and dementia 6-9. However, silent brain infarcts have not been specifically investigated in disadvantaged populations with very low levels of education using MRI 4. Studies evaluating the profile of cognitive deficits associated with silent cerebrovascular lesions in populations with low levels of education are also relevant because lower education influences cognitive reserves to a certain degree and may lead to greater cognitive deficits associated with brain lesions, whereas individuals with higher levels of education are expected to exhibit better sustained cognitive performance when suffering the same degree of brain damage 10,11.

In the present community-based study, we acquired structural MRI scans in a population-based sample of 250 elderly (66 to 75 years old) subjects without dementia or other neuropsychiatric disorders, including a high proportion of individuals with very low levels of education (4 years or less). This sample was recruited from a circumscribed, economically disadvantaged catchment area of Sao Paulo, Brazil. We determined the frequency of silent brain infarcts in this sample population and investigated the profile of cognitive deficits associated with those brain lesions. We predicted that the frequency of silent brain infarcts would be substantially high and more prevalent in subjects with fewer years of formal education.

## METHODS

The study received approval from the local Committee for Ethics and Research of the Faculty of Medicine, University of Sao Paulo (#0450/05). Written consent was obtained from all subjects.

Participants were selected from a community-based sample of elderly individuals recruited for the Sao Paulo Ageing and Health (SPAH) study 12, an epidemiological investigation aimed at determining the prevalence of dementia, other mental disorders, and their risk factors 12. Residents aged 65 or older from predefined census sectors of an economically disadvantaged area of Sao Paulo were recruited to undergo a cognitive evaluation using the protocol developed by the 10/66 Dementia Research Group 12-14. This protocol included a structured neurological assessment, a structured cardiological evaluation, the Geriatric Mental State (GMS) (a standardized psychiatric interview), and the Community Screening Instrument for Dementia (CSI-D) 12. The CSI-D consists of a 32-item cognitive test administered to the participant (approximately 20 minutes) and a 26-item informant interview that includes items inquiring about the participant’s daily functioning and general health (approximately 15 minutes) 15. Three summary scores are generated from the CSI-D, including the COGSCORE, an item-weighted summary score from the participant’s 32-item cognitive test (seven-item object denomination, four-item object definition, two verbal category fluency tasks, word repetition, identification of a famous person, temporal and spatial orientation, three orders, three-word recall, six-chunk story recall, two drawings of intersecting circles and pentagons) that also incorporates the Consortium to Establish a Registry for Alzheimer’s Disease (CERAD) animal naming verbal fluency task and the modified CERAD 10 word-list learning task with delayed recall 16; the informant score (RELSCORE), which is an unweighted total score from the informant interview; and the discriminant function score (DFSCORE), which is a weighted score combining COGSCORE and RELSCORE 17. The SPAH composite cognitive algorithm was used to exclude subjects with dementia.

Subjects also underwent a short (approximately 10 minutes) evaluation using the Short Cognitive Performance Test (SKT), which was validated in Brazil by Flaks et al. 18, to briefly document the cognitive performance of subjects on the day of MRI scanning. The SKT permits a rapid evaluation of overall cognitive performance and performance in specific domains, such as memory, attention and automatic inhibition, with higher scores indicating a more severe cognitive impairment 19. Thus, the measures of cognitive performance used in the present investigation included total SKT scores, SKT-based attention and memory subscores, total COGSCORE, and category verbal fluency scores. In addition, IQ estimates were obtained for all subjects who underwent MRI scanning with the Wechsler Abbreviated Scale of Intelligence (WASI) 20.

All subjects were also ranked according to their cardiovascular risk using the 10-year Framingham Risk Score for Coronary Heart Disease Risk (FRS), which is a composite index comprising five clinical factors (age, blood pressure, diabetes mellitus, smoking status, and cholesterol levels) 21,22. Subjects were classified into three subgroups according to their degree of cardiovascular risk (10-year risk): low-risk (<10%), medium-risk (10 to 20%), and high-risk (>20%) 22.

From the initial SPAH databank (n=2,072), we excluded all subjects with a diagnosis of dementia (n=105), subjects aged greater than 75 years at the time of recruitment for MRI scanning (n=996) and subjects with a history of major neurological disorders (such as epilepsy and Parkinson’s disease) or lifetime diagnosis of major depressive disorder according to the International Statistical Classification-10th revision criteria (ICD-10) (n=52). Eight hundred twelve potentially eligible individuals were identified after excluding individuals with missing clinical data (n=107). The remaining potentially eligible subjects were contacted by telephone to assess the presence of contra-indications for MRI scanning and to exclude individuals who had a previous history of severe head trauma. We failed to contact 103 subjects. Of the subjects who were contacted, we excluded 206 subjects (132 females and 74 males) who fulfilled the above exclusion criteria; thus, 503 subjects were invited to undergo the brain imaging session. After excluding illiterate subjects (i.e., individuals who could not write or read a simple message/letter) and excluding additional subjects due to budget constraints, only 306 of the 503 available individuals were invited to undergo the MRI scanning session. Fifty-two of the invited subjects refused to participate in the study, resulting in 254 dementia-free elderly subjects between 66–75 years old (female/male [139/115]) who underwent MRI scanning. We excluded illiterate subjects because the SKT-based cognitive performance indices that we acquired on the day of MRI scanning may have limited the validity of the results obtained from these individuals 18.

Schooling data were extracted from the SPAH database. We considered subjects to have 4 years of education if they had completed the 4th grade, 8 years if they had completed the 8th grade, 11 years if they had completed high school, and 15 years if they had completed university. If information on the participant’s education was not available, the number of years until he/she exited the education system was used as the estimate of the mean number of years of education. Current socioeconomic status was evaluated using Associação Brasileira de Anunciantes criteria 23.

No significant differences in age (t-test=1.354, *p*=0.176), prevalence of hypertension (Fishers exact test, *p*=0.480) or prevalence of diabetes (Fishers exact test, *p*=0.089) were observed between the individuals who underwent MRI scanning (n=254) and the eligible individuals who were not included in the study (n=558). The gender distribution was significantly different between the two groups (Fisher’s exact test, *p*=0.003), with a larger proportion of males in the group that underwent MRI scanning than the potentially eligible individuals who were not included in the study (45.3% *versus* 34.2%). The studied group also displayed a larger number of years of formal education (3.49±3.28 years) then the individuals who were not included in the investigation (2.15±2.74 years) (t-test=-6.038, *p*<0.01).

MRI datasets were acquired using a 1.5-T General Electric Signa LX CVi scanner (Milwaukee, WI, USA) with the following acquisition protocol: a) a dual-spin echo sequence of 120 transaxial slices across the entire brain (axial PD/T2); b) a T2-weighted fast spin-echo transaxial sequence (88 slices); and c) a 3D Spoiled Gradient Recalled Acquisition sequence of 124 slices with a repetition time (TR)/echo time (TE) of 21.7/5.2 msec, a flip angle of 20 degrees, 220-mm field of view, 1.5-mm slice thickness, 1 measurement, and a 256×192 matrix. All MRI scans were examined by experienced radiologists who were unaware of the study aims, and they identified the presence, number and location of silent brain infarcts. Infarcts were detected as low-signal-intensity lesions on the spoiled gradient echo (SPGR) sequence and hyperintense lesions on the T2-weighted images. Vascular lesions that were 3 to 15 mm in diameter were classified as lacunae 24,25

The Statistical Package for Social Sciences (SPSS) for Windows (17.0) was used for the statistical analyses. Cognitive data obtained from elderly individuals with and without silent brain infarcts were compared using ANOVA (statistical significance set at *p*<0.05). For comparisons of each individual group, subjects were automatically excluded when values for the specific variable under evaluation were missing. For the overall sample of 254 elderly subjects, data for FRS scores (n=6), IQ (n=1), and COGSCORE (n=10) were missing.

## RESULTS

### Frequency of silent brain infarcts

Four of the 254 subjects assessed with MRI were excluded from the analyses due to artefacts during MRI scanning that prevented the accurate detection of gross brain lesions, resulting in a total of 250 individuals who were selected for the analysis. Fifty-seven individuals had at least one silent vascular-related lesion, yielding a prevalence of 22.8% (95% confidence interval: 17.7 to 28.5). Details for these lesions and their brain locations are provided in Table 1. An additional 4 subjects presented with meningioma and 2 had vascular malformations.

When we restricted the MRI data analysis to the subsample of individuals with very low levels of education (4 years or less, n=185), 25.41% (n=47) were presented at least one silent vascular-related lesion.

### Demographic and clinical differences between subjects with and without silent vascular-related brain lesions

After excluding individuals with meningioma or vascular malformations, the subgroup with silent vascular lesions (n=57) was older (*p*<0.001) and had lower levels of education than the subgroup with no brain infarcts (*p*=0.038) (Table 2). No significant differences in the gender distribution, current socioeconomic status and estimated IQ were observed between individuals with and without silent infarcts (Table 2). The proportion of individuals with a high 10-year cardiovascular risk (FRS>20%) was greater in the subgroup with silent vascular lesions in the brain than that in the subgroup without silent infarcts (Table 2).

When we restricted the analysis to the subsample of individuals with very low levels of education (4 years or less, n=185; 21 with no formal education), the subgroup with silent brain infarcts (n=47) was older than individuals without silent brain infarcts (72.43±3.41 *vs* 70.60±2.68, t=-3.749, *p*<0.001), and a trend towards a greater proportion of individuals with a high cardiovascular risk (55.3% *vs* 39.1%, *p*=0.079) was observed. No significant differences in gender distribution, current socioeconomic status and estimated IQ were observed between individuals in the subsample with very low levels of education who presented with or without silent infarcts.

### Comparison of cognitive performance between subjects with and without silent vascular-related brain lesions

The subgroup with silent vascular lesions performed significantly worse on all cognitive measures, except for the verbal fluency category (Table 3). Cohen’s d values were greater than 0.40 for all cognitive measures, except for the verbal fluency category (Table 3). Total raw SKT scores were not able to be transformed into norm values for 11 individuals with low estimated IQ values.

Based on the between-group differences in mean current age and level of education (Table 2), we repeated the analyses and used age and years of education as confounding covariates. The subgroup with brain lesions again performed significantly worse, based on their COGSCORE performances (*p*=0.014), total SKT scores (*p*=0.015), SKT-based memory subscores (*p*=0.004) and attention subscores (*p*=0.038).

Finally, we counted the number of individuals with a COGSCORE performance that was 1.5 standard deviations below the mean performance of all subjects between 66 and 75 years of age from the original SPAH sample. This analysis generated the criterion for mild cognitive impairment (MCI) based on the actual mean cognitive profile of the elderly population from which the present sample was drawn 26. Significantly more individuals (n=5) with silent vascular lesions who fulfilled the criterion (8.77%), as only 4 individuals with MCI were identified in the subgroup without such brain lesions (2.14%) (Fisher’s exact test, *p*=0.034). Four individuals who fulfilled the MCI criterion in the subgroup with silent brain infarcts (80%) and 4 subjects with MCI in the subgroup with no vascular brain lesions (100%) had less than four years of formal education.

## DISCUSSION

This study investigated the presence of silent brain infarcts detected by MRI scanning and their associated cognitive deficits in a relatively large sample of apparently healthy individuals aged 66-75 years who were recruited from an economically disadvantaged elderly population in Sao Paulo, Brazil. The frequency of silent brain infarcts in our sample was considerably high and represented approximately 23% of subjects from 66-75 years old. The frequency of silent brain infarcts reported in this study is at the higher end of the range of prevalence estimates reported in MRI studies conducted on populations from high-income countries 2,4. Moreover, our sample was restricted to individuals aged 65-75 years, whereas many previous studies included older subjects who may have a higher frequency of these lesions 27. Prevalence estimates in community-based samples with a mean age that is comparable to the ages included in our study have ranged from 8% to 20% 2,4,28. Thus, according to our study, the prevalence of silent brain vascular lesions in elderly subjects recruited in an economically disadvantaged urban centre may be substantially high.

The FRS has been shown to underestimate cardiovascular disease risk in socioeconomically disadvantaged individuals 29. Nevertheless, we detected a significant, direct relationship between silent brain infarcts and higher FRS in our economically disadvantaged cohort, which replicates findings from studies conducted in higher-income environments 28,30.

As predicted, the presence of silent brain infarcts was associated with significantly worse cognitive performance. Significant deficits were detected in memory and other cognitive domains, reinforcing the view that covert vascular lesions in elderly populations are related to deficits in several cognitive domains 31,32. Cohen’s d values were moderate (approximately 0.5) for the total and memory SKT scores and greater than 0.40 for the COGSCORE and the SKT attention subscore. These effect sizes were not modest, suggesting that cognitive deficits associated with silent brain infarcts in elderly subjects recruited in an economically disadvantaged urban centre should not be judged as subtle, as suggested by other researchers examining elderly subjects in higher-income environments 33,34.

Consistent with previous investigations conducted on other populations as well as populations in Brazil, we detected more vascular brain lesions in less educated individuals 35,36. The overall sample investigated in the present study predominantly had low levels of education, with a large proportion of individuals (75.82%) having less than four years of formal education. The frequency of silent vascular-related brain infarcts surpassed the level of 25% in our sample when the inspection of MRI data was restricted to individuals with very low levels of education (4 years or less). This frequency of silent brain infarcts is greater than the average values reported in a number of studies conducted on populations with higher mean levels of education 2,4,28. Therefore, the prevalence of silent brain infarcts in individuals with very low levels of education detected in our study may be disproportionally large, which is consistent with the known unfavourable association between lower levels of education and cardiovascular risk factors 37. Since potentially eligible subjects who did not undergo MRI scanning had a significantly lower mean number of years of formal education, our prevalence rates of silent brain infarcts may have been even higher if we had examined the illiterate individuals who were not included in the study. Notably, the majority of individuals who fulfilled our MCI criterion presented silent brain infarcts and had very low levels of education (less than four years). This outcome is consistent with the hypothesis that reduced cognitive reserves increase the risk of clinically significant cognitive deficits in the presence of brain damage 10.

The preferential location of silent brain infarcts in the basal ganglia in our study is highly consistent with the findings of previous studies 3 and supports the hypothesis that this brain region exhibits greater vulnerability to cerebrovascular-related damage and the associated cognitive deficits in multiple domains 34. Based on recent tractography MRI studies using diffusion tension imaging, even small silent vascular basal ganglia lesions may affect the function of large-scale cortical white matter networks, providing a potential explanation for the emergence of the association between cognitive deficits in multiple domains with silent basal ganglia infarcts 32. Moreover, resting-state functional MRI investigations have revealed aberrant patterns of functional connectivity in both intra- and between brain networks in patients with vascular basal ganglia lesions, which are directly proportional to the degree of cognitive impairment 31. Subgroups with and without silent brain infarcts in our study did not exhibit differences in category fluency scores. This lack of difference is not likely to be due to compensatory brain mechanisms that are recruited to sustain fluency performance in subjects who presented cerebral lesions, because other studies have reported verbal fluency deficits in elderly subjects with silent brain infarcts 33,38. Category verbal fluency scores were relatively modest in both elderly subgroups (possibly due to the influence of the overall low level of education in the sample) 39. The scores obtained in our sample are lower than the scores reported with the same version of the task applied to populations of elderly individuals without dementia who were examined in Latin-American countries, India and China 40. Therefore, floor effects may have prevented us from detecting subgroup differences in this task.

Moreover, the subgroup with silent vascular-related brain lesions in our study was older than the subgroup without these lesions. However, this age difference was not likely to have weakened our reported association between significantly worse cognitive performance and silent vascular brain lesions for two reasons. First, both subgroups had mean current ages of greater 70 years, and the between-group difference in mean age values was less than 2 years. Second, measures of cognitive performance remained significantly different between groups when we repeated the statistical analyses by entering the current age as a confounding covariate.

We must acknowledge that our investigation was restricted to documenting the presence of sizeable brain infarcts through a visual inspection of morphological scans acquired with a 1.5-T scanner. We did not assess the presence of changes of potentially lesser cerebrovascular disease severity in the brain, including white matter hyperintensities (which are thought to reflect microvascular injuries in elderly populations) 41-43 or microinfarcts (which may not be clearly detectable using a 1.5-T MRI scanner) 44,45. Therefore, the prevalence rates of silent cerebrovascular disease markers may have been even larger in our elderly population if we had been able to assess the full range of these markers. Another limitation is that not all eligible individuals were evaluated with MRI due to the exclusion of illiterate subjects and budget constraints. Thus, the figures reported in this study cannot be considered true population-based prevalence estimates of silent brain infarcts.

Our results obtained from a sample of predominantly poorly educated elderly individuals from an economically disadvantaged urban region indicate that covert cerebrovascular disease is frequent. As suggested by previous findings, covert vascular brain damage has meaningful consequences and significantly contributes to deficits in cognitive performance that may be considered related to aging in the absence of MRI data 4. Our findings reinforce the need to carefully examine the potential deleterious effects of vascular brain lesions in poorly educated elderly individuals from economically disadvantaged environments. Future longitudinal studies are needed to investigate the long-term prognosis and rates of conversion to dementia in these individuals.

## SOURCES OF FUNDING

This research was funded by the Wellcome Trust, UK (GR066133MA) and by FAPESP (2004/15336-5, 2012/50329-6 and 2013/03231-3), Brazil. PS was supported by FAPESP-Brazil (2013/03231-3). GFB, MS, PRM, and TCTFA were supported by CNPQ-Brazil.

## AUTHOR CONTRIBUTIONS

Busatto GF conceived the study and participated in study design. Squarzoni P analyzed and interpreted the data and drafted and revised the manuscript. Tamashiro-Duran JH, Duran FL, Leite CC, Wajngarten M, Scazufca M, Menezes PR, Lotufo PA and Alves TC analyzed and interpreted the data and revised the manuscript. All authors read and approved the final version of the manuscript.

## Figures and Tables

**Table 1:** Location of silent vascular brain lesions (n=57).

Brain location	Number of subjects	%
Basal ganglia/thalamus	26	45.61
White matter	9	15.79
Basal ganglia/thalamus/cerebellum/brainstem	4	7.02
Cerebellum	4	7.02
Basal ganglia/thalamus/cerebellum/brainstem/frontal cortex	1	1.75
Basal ganglia/thalamus/white matter	1	1.75
Cerebellum/brainstem	3	5.26
Basal ganglia	2	3.51
Parietal cortex	2	3.51
Basal ganglia/thalamus/white matter/cerebellum/brain stem	1	1.75
Basal ganglia/thalamus/white matter/cerebellum/brain stem/occipital cortex	1	1.75
Frontal cortex	1	1.75
Occipital cortex	1	1.75
Temporo-occipital cortex	1	1.75

**Table 2:** Demographics, estimated intelligence and medical characteristics of the participants in the subgroups with and without silent vascular lesions.

	With vascular brain lesions (n=57)	Without vascular brain lesions (n=187)	*p*
**Men**	25 (43.9%)	88 (47.1%)	0.672^a^
**Mean years of education (±SD)**	3.46 (±2.66)	4.54 (±3.63)	0.038^a^
**Mean age (±SD) in years**	72.11 (±3.36)	70.51 (±2.57)	<0.001^a^
**Hypertension (%)**	39 (68.4%)	114 (61.0%)	0.308^b^
**Diabetes (%)**	19 (33.3%)	49 (26.3%)	0.304^b^
**High cardiovascular risk (%)** *	30 (52.6%)	67 (35.8%)	0.034^b^
**Estimated IQ (±SD)**	75.84 (±11.33)	76.92 (±10.19)	0.497^a^

SD: *standard deviation; IQ = estimated coefficient of intelligence.*

* As determined by the Framingham Coronary Heart Disease Risk score (FRS). Subjects were classified as low-risk (<10%), medium-risk (10 to 20%), and high-risk (>20%).

a Unpaired t-tests;

b Chi-square tests (Pearson).

**Table 3:** Comparisons of the performance of individuals with and without silent vascular brain lesions on cognitive tests.

	With vascular brain lesions (n=57)	Without vascular brain lesions (n=187)	Cohen’s d	*p*^a^
**COGSCORE (±SD)**	28.26 (±3.18)	29.38 (±0.81)	0.42	0.002
**Verbal fluency (±SD)**	13.75 (±5.07)	14.51 (±4.60)	0.16	0.260
**Total SKT score (±SD)**	8.75 (±6.19)	6.14 (±4.15)	0.49	<0.001
**Memory SKT subscale (±SD)**	1.70 (±1.89)	0.90 (±1.08)	0.51	<0.001
**Attention SKT subscale (±SD)**	7.27 (±4.90)	5.24 (±3.82)	0.45	0.002

SD: *standard deviation*.

a Unpaired t-tests
